# Urine Cytological Diagnostics: Possibilities and Limitations—A 25-Year Review and Overview at Hannover Medical School

**DOI:** 10.3390/clinpract15120234

**Published:** 2025-12-12

**Authors:** Soudah Bisharah, Mieke Raap, Mahmoud Abbas

**Affiliations:** 1 Independent Researcher, Heidering 18, 30625 Hannover, Germany; soudah.bisharah@gmail.com; 2 Institut für Pathologie, Medizinischen Hochschule Hannover (MHH), Carl-Neuberg-Straße 1, 30625 Hannover, Germany; raap.mieke@mh-hannover.de; 3 Gerhard-Domagk-Institut für Pathologie, Universitätsklinikum Münster, Domagkstrasse 17, 48149 Münster, Germany

**Keywords:** urine cytology, urothelial carcinoma, diagnostic accuracy, cytomorphology, cytological features, sensitivity and specificity

## Abstract

Background: Urine cytology is a highly effective, straightforward, and cost-efficient diagnostic tool for identifying neoplastic and non-neoplastic changes in the bladder, ureter, and renal pelvis. The aim of this study is to demonstrate the high sensitivity and specificity of urine cytology in detecting a wide range of urothelial lesions, including metastatic involvement. Material and Methods: Urine cytology was performed on 9639 cases between 2000 and 2025. The samples, collected from patients, were processed at the Institute of Pathology. Cytological slides were prepared using cytocentrifugation and stained with May–Grünwald–Giemsa (MGG) and Papanicolaou stains. The cytological findings were classified according to WHO, 2004 compared with histological specimens. Additionally, selected cases underwent immunohistochemical and molecular analyses. All samples were anonymized and retrospectively analyzed following the guidelines and regulations of the local ethics committee. Results: Of the total cases, 7051 were classified as benign, 1269 as malignant, and 88 as normal findings. Insufficient material was obtained in 336 cases. No complications were reported during sample collection or processing. The concordance with histological findings for neoplastic lesions was over 96%, with a false-negative rate of 1.84%. The diagnostic methods demonstrated a sensitivity of 90.7% and a specificity of 96.64%. Among the 6956 cases analyzed, 3139 were women (45.13%) and 3817 were men (54.87%). Conclusions: The diagnostic value of urine cytology in representative material is relatively high in assessing both the presence or absence of malignancy and, when applicable, the tumor grade. This large 25-year single-center review demonstrates that urine cytology retains high sensitivity and specificity for the detection of urothelial malignancy, particularly high-grade disease. However, the atypical category remains a major diagnostic challenge and contributes substantially to false-positive results.

## 1. Introduction

Cancers of the urinary bladder are the 10th most common malignancies worldwide and the 13th most common cause among all cancer-related deaths [1]. The cytological examination of urine—whether spontaneous, voided, or catheterized urine—is a valuable method for diagnosing both non-neoplastic and neoplastic cellular changes in the urothelium. It is particularly effective for the early detection of cancers in the urinary bladder, urinary tract, and renal calyceal system, with high specificity for detecting high-grade urothelial carcinoma [2]. Early diagnosis allows for timely treatment, significantly improving recovery outcomes. Urine cytology has become increasingly important in evaluating the bladder, ureter, and renal pelvis, offering significant utility in differentiating inflammatory lesions from neoplasms [3]. In recent years, the Paris System for Reporting Urinary Cytology (TPS) has provided a standardized framework focused on the detection of high-grade urothelial carcinoma. TPS emphasizes nuclear atypia, nuclear-to-cytoplasmic ratio, and specific cytomorphological criteria, aiming to reduce overuse of the ‘atypical’ category and improve reproducibility. In addition to cytomorphology, molecular techniques such as FISH (UroVysion) and immunohistochemistry (e.g., CK7, CK20, p63, GATA3) enhance diagnostic accuracy, especially in challenging or atypical cases. However, urinary tumor markers (e.g., NMP22, BTA) are not yet recommended for routine use due to limited sensitivity and cost-effectiveness. According to the 2022 WHO Classification of Tumors of the Urinary System and Male Genital Organs [4], cytological evaluation remains crucial for early detection of urothelial neoplasia. Accurate interpretation of cytological findings depends on sample quality, cytopathologist expertise, and the integration of clinical and anatomical context.

## 2. Material and Methods

### 2.1. Case Selection

Urine cytology was performed on 9639 cases between January 2000 and January 2025. All samples were processed at the Institute of Pathology. The cases were retrospectively analyzed in accordance with the guidelines and regulations of the local ethics committee, with all samples anonymized.

### 2.2. Sample Collection

Urine samples were obtained using various methods, depending on clinical indications. Average volume per sample was 10–50 mL depending on the method.

**Figure 1:**
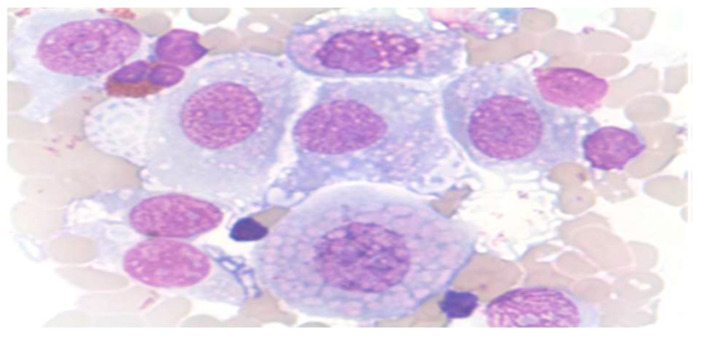
Normal urothelial cells in urine sample. (MGG, obj. ×40).

**Figure 2:**
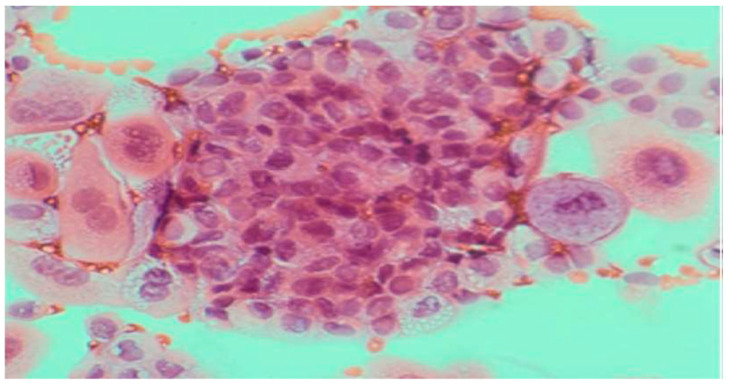
Rinsing (Irrigation) urine without atypia. (Papanicolaou, obj. ×20).

**Figure 3:**
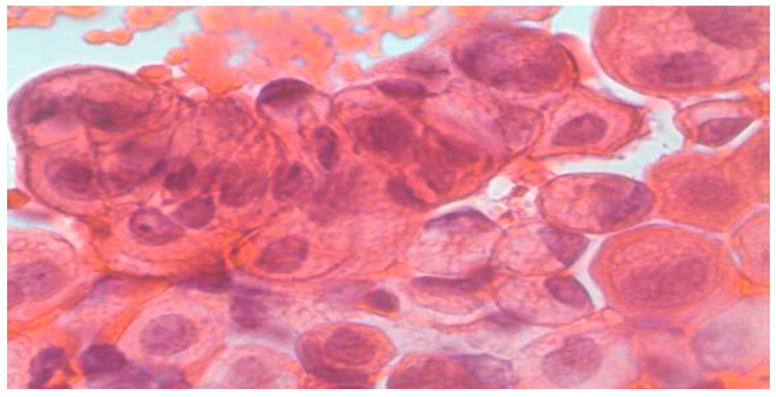
Urothelial cells in catheterized urine without changes. (Papanicolaou, obj. ×40).

- Spontaneous urine, ideally from the second morning void, was preferred for standard analysis (Figure 1).
- Catheterized urine was frequently used and often contained epithelial formations from the urethra (Figure 2).
- Voided urine helped in localizing tumors within the urinary tract (Figure 3).
- Inadequate sample is defined when there are <500 cells, or heavily bloody or granular, >50% degenerated.
- Additional collection methods included:
  ○ Timed urine collection (4–24 h)
  ○ Midstream urine (primarily for infection diagnosis)
  ○ Brush smear technique
  ○ Ileum conduit samples
  ○ Suprapubic bladder puncture

### 2.3. Slide Preparation and Staining Techniques

- For each case, four cytocentrifuge slides were prepared using a cytocentrifuge by Cytospin (Kirchlengern, North Rhine-Westphalia, Germany) at 800 rpm for 10 min. In cases of bloody or viscous samples, sediment smears were also prepared.
- Staining protocols included:
- May–Grünwald–Giemsa (MGG)
- Papanicolaou stain
- Additionally, phase contrast microscopy was performed using unstained slides without coverslips. Special stains were applied in selected cases to address specific diagnostic questions.

### 2.4. Cytological Classification and Ancillary Techniques

- Cytological atypia was classified according to the WHO 2004 [5] classification system (Table 1) and correlated with available histological specimens for diagnostic accuracy.
- To improve diagnostic accuracy in suspected metastatic cases, additional immunohistochemical and molecular techniques were employed.

### 2.5. The Atypical Urothelial Cells (AUCs)

These are cells that are not clearly benign (reactive/inflammatory) but do not meet all criteria for a neoplasm.

Common features: mild to moderate nuclear enlargement, irregular nuclear shape, coarse chromatin, slightly increased N/C ratio, no definitive malignant features.

Purpose: to identify cases that are not clearly negative but also not definitively malignant, allowing for appropriate clinical follow-up.

### 2.6. Sensitivity and Specificity Calculation

- Positive cases: histologically confirmed low- or high-grade urothelial neoplasms.
- Negative cases: cytologically normal and inflammatory/reactive lesions.
- Excluded from sensitivity calculation: insufficient samples (n = 336).
- False-negative cases: cytologically non-malignant but histologically confirmed neoplasms.
- False-positive cases: cytologically atypical or suspicious but histologically benign.

## 3. Results

Of the 9639 urine cytology cases analyzed, 88 cases were classified as cytologically normal, and 336 samples were deemed insufficient for diagnosis (Table 2). Among 7051 cases with inflammatory or reactive cytological changes, 130 were later confirmed as malignant based on histological follow-up, resulting in a false-negative rate of 1.84%.

Of the 895 cases showing atypical urothelial changes, 451 were histologically confirmed as malignant, while 245 showed no evidence of neoplasia, yielding a false-positive rate of 35.2% (Table 3). Histological correlation was unavailable for 199 atypical cases. Importantly, among the cytologically normal cases, no false-negative results were observed.

A detailed analysis of the 130 false-negative cases revealed that 63.86% were diagnosed histologically as high-grade urothelial carcinoma (G2–G3), frequently associated with ulceration and marked inflammation. An additional 9.23% represented carcinoma in situ. Overall, 73.09% of false-negative cases corresponded to high-grade urothelial lesions (Table 4), emphasizing the diagnostic challenge in the presence of extensive inflammation or ulceration. We found a sensitivity of 90.7% and a specificity of 96.64% in detecting various urinary bladder lesions.

**Cytomorphological Summary** (moved from main results to improve readability, can also be provided in Supplementary Materials)

- Normal urothelial and non-urothelial cells: umbrella cells, intermediate, basal, squamous, renal tubular, seminal vesicle, and urethral gland cells (Figure 1, Figure 2 and Figure 3, and Figures S1–S4).
- Reactive/inflammatory changes: catheterization, metaplasia, post-radiotherapy, infections, cytostatic therapy, urothelial hyperplasia, crystal-associated changes, cystitis variants, metanephric metaplasia, malakoplakia, inflammatory pseudotumors (Figures S5–S12).
- Atypical, dysplastic, and neoplastic changes: AUC, flat neoplasms (dysplasia, CIS), benign and malignant papillary neoplasms, invasive carcinoma, metastatic tumors (Figure 4 and Figure 5, and Figures S13–S24,).

**Figure 4:**
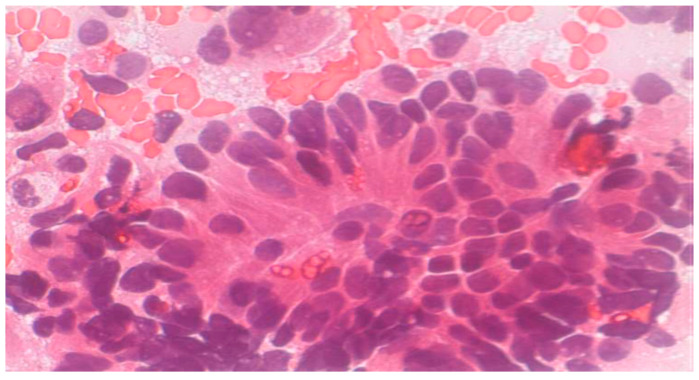
Metastasis of colon carcinoma. (Papanicolaou, Obj, ×40).

Metastatic Tumors (Table 5):

Colorectal adenocarcinoma: elongated, mucin-producing neoplastic glands with hyperchromatic nuclei, often in inflammatory or necrotic background (Figure 4).

**Figure 6:**
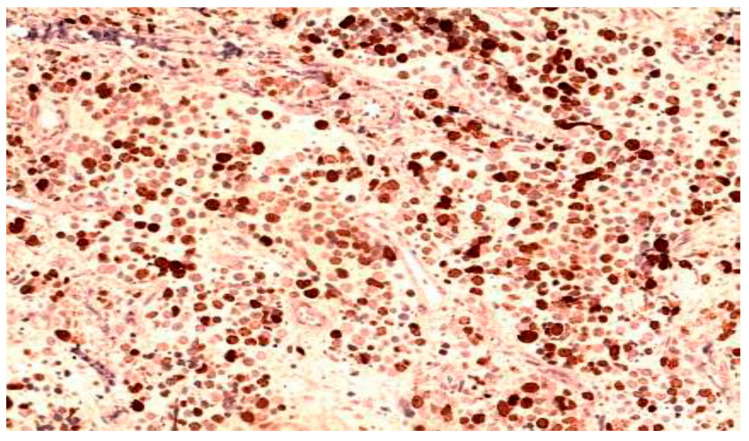
Neuroendocrine carcinoma (Chromogranin A, Obj., ×20).

Neuroendocrine tumors: small to medium-sized hyperchromatic cells; confirmed via immunohistochemistry (e.g., chromogranin A; Figure 6).

Lymphoma, melanoma: detectable with immunohistochemical and molecular techniques.

**Explanation of total cases (n = 1861) in Table 5:** The total number of cases listed (n = 1861) represents the sum of all cytologically and histologically confirmed malignancies, including urothelial neoplasms and metastases. This includes 1269 cases with cytologically diagnosed malignant urothelial lesions (Table 2), 451 cases with atypical urothelial cells (AUC) that were later confirmed as malignant on histology (Table 3), 130 cases with inflammatory or reactive cytology that were found to harbor neoplasia on histology (Table 4, false negatives), and 11 cases of metastatic tumors (Table 5). This cumulative total ensures that all confirmed neoplastic events are accounted for.

- **In Summary** (Table 6)

**Case distribution and derivation of sensitivity and specificity:**

- Total cases: 9639; analyzed cases with sufficient cytology: 9303 (after excluding 336 cases with insufficient or inappropriate material).
- Cytologically normal: 88; insufficient: 336.
- Inflammatory/reactive changes: 7051; false negatives (FN) among these were 130 (1.84%), leaving 6921 true non-malignant cases.
- Atypical urothelial changes (AUCs): 895; of these, 451 were later confirmed as malignant, 245 showed no evidence of neoplasia (false positives, FP = 245), and 199 lacked histological correlation.
- Total number of non-malignant (inflammatory/reactive) samples: 6921. Atypical cellular changes were observed in 895 samples, of which 245 showed no malignancy (false positives, FP = 650).
- Most false-negative cases (63.86%) were HGUC (G2–G3), often masked by inflammation or ulceration.
- Malignant urothelial changes (TP) were confirmed in 1269 cases.

Calculation of diagnostic performance:

- Sensitivity = TP/(TP + FN) = 1269/(1269 + 130) = 90.7%
- Specificity = TN/(TN + FP) = 6921/(6921 + 245) = 96.64%

## 4. Discussion

Urine cytology is a non-invasive and straightforward method for detecting urothelial lesions, known for its high sensitivity and specificity in identifying high-grade urothelial carcinoma (HGUC), as first described by Papanicolaou GN and Marshall VF [3]. Reid et al. [6] (2012) reported a specificity of 85–90% for urine cytology, while Mowatt et al. [7] (2010) observed a sensitivity of 44% in detecting bladder cancer, with a specificity of 96%. Piaton et al. [8] (2005) reported a mean sensitivity of approximately 50% for detecting urothelial carcinoma. In our retrospective study, we found a sensitivity of 90.7% and a specificity of 96.64% in detecting various urinary bladder lesions. The unusually high sensitivity of 90.7% in our cohort may be partly explained by the high proportion of high-grade cases (63.86% HGUC among histologically confirmed tumors). High-grade tumors are known to shed more atypical cells and therefore are more easily detected cytologically compared to low-grade lesions. Another contributing factor may be the long-term expertise of a specialized cytopathology center affiliated with a university hospital and serving as a reference center for malignant cases. Another contributing factor may be the long-term expertise of a specialized cytopathology center affiliated with a university hospital and serving as a reference center for malignant cases. A high false-positive rate in AUCs (35.2%) may reflect overlap with reactive changes and emphasizes the importance of histologic follow-up. A major challenge in urine cytology is the interpretation of cases with atypical changes. Studies have reported the prevalence of atypical changes ranging from 1.9% to 23.3%. Mokhtar GA et al. [2] (2010) and Muus Ubgas et al. [9] (2013) reported that 23% of cytologic specimens exhibit some degree of atypia without meeting the criteria for urothelial carcinoma. In our study, 9.3% of cases showed atypical urothelial changes, of which 69.38% were later diagnosed as low or high-grade urothelial neoplasms. Notably, 1.8% of samples with inflammatory lesions were later found to harbor malignant urothelial lesions in histological biopsies. Interestingly, 63.86% of these cases were high-grade urothelial neoplasms (G2-G3), which contrasts with previous reports suggesting a high sensitivity for detecting high-grade lesions. Poor cell shedding, necrosis, and masking by inflammation are probable biological explanations.

We hypothesize that ulcerative inflammatory lesions, which contain numerous inflammatory cells and debris, may obscure malignant cells, making diagnosis more challenging. Our findings align with those of Lee et al. [10] (2016), who reported that 36.8% of apparent false-negative urine cytology results could not be prevented even after retrospective review by two experienced cytopathologists. This was despite processing techniques after cytocentrifugation that normally eliminate red blood cells and debris and reduce the number of inflammatory cells. In these cases, malignant cells were either poorly preserved or obscured by inflammation. Additionally, they noted that high-grade urothelial carcinomas (HGUCs) in these cases did not shed sufficient malignant cells into the urine for detection.

To improve the sensitivity of urine cytology in detecting hidden urothelial neoplasms, some authors, including Chang et al. [11] (2015), have proposed ancillary techniques such as immunocytochemistry and FISH analysis.

In summary, urine cytology plays a significant role in assessing the nature and grade of urothelial neoplasia. However, its diagnostic accuracy relies on two key factors: the availability of relevant clinical information and the experience of the cytopathologist. Despite its usefulness, urine cytology presents several challenges and potential pitfalls. These include changes induced by BCG therapy, alterations in samples from patients with permanent catheters, degenerative cellular changes, and the presence of Polyomavirus infections, all of which can complicate interpretation and potentially lead to diagnostic uncertainty.

**Study Limitation:**

- Sample heterogeneity (voided, catheterized, timed, ileal conduit, etc.) may affect cellular yield, contamination, and morphology, influencing sensitivity, specificity, and false-positive/negative rates over this 25-year period.
- Extensive cytomorphological descriptions were summarized in a dedicated section to improve readability.
- Diagnostic criteria for AUCs and differentiation from low/high-grade lesions are now explicitly described to enhance interpretative clarity.
- Of the 895 AUC cases, 199 (22.2%) lacked histological follow-up. Excluding these cases from false-positive calculations may lead to selection bias and overestimation of diagnostic accuracy. This limitation must be considered when interpreting the false-positive rate of 35.2% in the atypical category.

Although the WHO 2004 classification was used in the present study due to its historical design, this represents an important limitation. Reclassification according to TPS 2022 would likely alter the distribution of atypical cases and false-positive rates and might improve comparability with contemporary studies.

- Retrospective design, incomplete histology, heterogeneous sample collection, potential selection bias over the 25-year period.
- Future directions: integration of molecular diagnostics and AI-assisted cytology may enhance sensitivity and reduce interpretive variability.

## Figures and Tables

**Figure 5:**
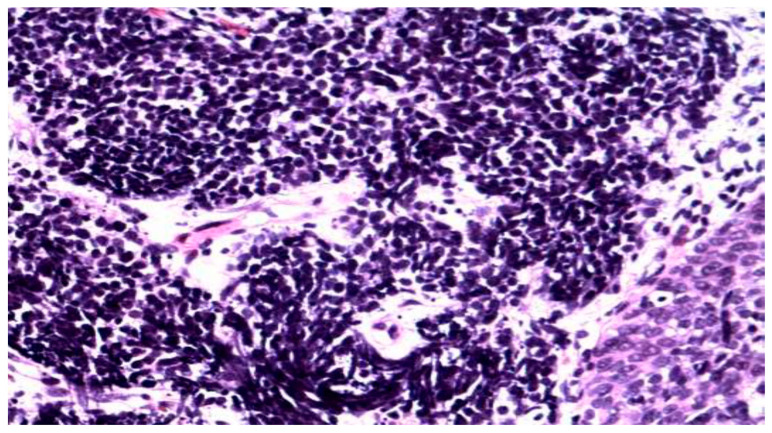
Neuroendocrine carcinoma (Papanicolaou Obj., ×20).

**Table 1:** The cytological atypia according to WHO, 2004.

Cytology	G1	G2	G3
Cell polymorphy	−/+	+	++/+++
Cell size	+	++	+++
Nucleous-plasma-relation	+	++	+++
Nucleus size	+	++	+++
Nuclear shape	round-oval	irregular	pleomorph
Nuclear polymorphism	+	+	+++
Chromatin	fine	irregular	hyperchromatic
Nucleoli	−	−	++
Mitosen	−	−/+	++
Umbrella cells	++	+	−/++
Cell groups	++	+/++	−/+++

(+) Mild (++) moderate (+++) severe.

**Table 2:** Cytological diagnosis of urine samples 2000–2025 (n = 9639). F = 3139 (45.12%), M = 3817 (54.87%).

Cytological Diagnosis	n	%
Non-sufficient material	336	3.47
Normal urine samples	88	0.90
Inflammatory-reactive changes	7051	73.15
Atypical urothelial changes	895	9.30
Malignant urothelial changes	1269	13.18
Total	9639	100%

**Table 3:** Histological diagnosis of 895 urine samples with atypia.

Histological Diagnosis	n	%
Low-grade samples (G1)	75	8.38
High-grade samples (G2 and G3)	89 + 162 = 251	28.04
Carcinoma in situ	110	12.29
PUNLMP	15	1.68
Urine samples without histological atypia or malignancy	245	27.38
Urine samples without histology	199	22.23
Total	895	100%

**Table 4:** The histological diagnosis from urine samples with inflammatory and reactive changes (n = 130).

Histological Diagnosis	n	%
Low-grade (G1)	33	25.38
High-grade (G2 and G3)	83	63.86
Carcinoma in situ	12	9.23
PUNLMP	2	1.53
Total	130	100

**Table 5:** Both primary and secondary (metastatic) lesions were identified in urine samples (n = 1861).

Diagnosis	n	%
Urothelial neoplasia	1850	99.40
Metastasis	11	0.60
Renal cell carcinoma	2	
Colonic carcinoma (Figure S12)	2	
Prostata carcinoma	2	
Chondrosacoma	1	
Vaginal carcinoma	1	
Malignant melanoma	1	
Gastric adenocarcinoma	1	
Pancreas carcinoma	1	
Total	1861	100%

**Table 6:** Contingency table comparing cytology with histology (n = 9,303).

Cytology\Histology	Malignant (TP + FN)	Non-Malignant (TN + FP)	Total
Malignant (TP)	1269	245 (false positives)	1514
Non-Malignant	130 (false negatives)	6921	7051
Total	1399	7166	8565

## Data Availability

All data are available and will be provided upon request.

## Supplementary Materials

The following supporting information can be downloaded at: https://www.mdpi.com/article/10.3390/clinpract15120234/s1, Figure S1: Superficial cells (umbrella cells). (MGG, obj., ×40); Figure S2: Intermediate urothelial cells. (Papanicolaou, obj., ×20); Figure S3: Basal urothelial cells. (Papanicolaou, obj., ×40); Figure S4: Renal tubular epithelial cells. (Papanicolaou, obj., ×20); Figure S5: Macrophages and Urothelzellen with cytoplasmic vacuoles after administration of contrast medium. (Papanicolaou, obj., ×20); Figure S6: Urothelial changes after radiotherapy. (Papanicolaou, obj., ×40; Figure S7: A: Decoy cells by Polyomavirus (Papanicolaou, obj., ×40). B: Immunohistochemistry: SV40-positive (obj., ×20); Figure S8: a,b: Conduit urine (Papanicolau, obj., ×10); Figure S9: Urine changes after BCG-therapy. (Papanicolaou, obj, ×20.); Figure S10: a, b: Urocystitis cystica et glandularis Brunn’s cell nests (a: Papanicolau; b: HE obj., ×40/obj., ×10); Figure S11: Malakoplakia (MGG obj., ×40); Figure S12: Inflammatory changes of urothelial cells (MGG, obj., ×40); Figure S13: a,b,c: Urothelial dysplasia. (Papanicolaou, obj., ×20); Figure S14: Urothelial carcinoma in situ (CIS). (Papanicolau, obj., ×20); Figure S15: Papilloma. (MGG, obj., ×20); Figure S16: Urothelial carcinoma, G1 (Papanicolau, obj., ×20/×40); Figure S17: Urothelial carcinoma, G2 (Papanicolau, obj., ×40); Figure S18: Low-grade urothelial neoplasma (MGG, obj., ×20); Figure S19: Spontaneous urine, papillary low grade urothelial neoplasma, G1 (Papanicolau, obj., ×20); Figure S20: UB ca., G3 (MGG, obj. ×20); Figure S21: Urothelial carcinoma, G2 (Papanicolau, (obj., ×40); Figure S22: Urothelial carcinoma of the bladder. (Papanicolaou, obj., ×40); Figure S23: Urothelial carcinoma G3 (Papanicolau, (obj., ×20); Figure S24. Urothelial carcinoma., G3 (Papanicolau, (obj., ×20).
