# Supplementary material for: Urine Cytological Diagnostics: Possibilities and Limitations—A 25-Year Review and Overview at Hannover Medical School
Source: Clin Pract. 2025 Dec 12;15(12):234. doi: 10.3390/clinpract15120234 (PMC12732187; doi:10.3390/clinpract15120234)
Supplement: Supplementary file 1 [file clinpract-15-00234-s001.zip › clinpract-3994337-supplementary.pdf]

clinics and practice

Article

Urine Cytological Diagnostics: Possibilities and Limitations—A 25-Year Review and Overview at Hannover Medical School

To better contextualize these diagnostic findings, a detailed description of the cytomorphological features of normal, reactive, and neoplastic cells in urine sediment is provided below.

1. Cytomorphological Appearance of Normal Urothelial and Non-Urothelial Cells

Superficial transitional cells (umbrella cells) are large, polygonal or elongated, with centrally located vesicular nuclei and abundant wedge-shaped cytoplasm. Binucleation is common; multinucleation may occasionally be observed (Fig. S1). Intermediate cells often appear pear-shaped or tailed, with vesicular nuclei and extended cytoplasm (Fig. S2). Basal cells are typically cuboidal, cylindrical, or wedge-shaped, with small, oval nuclei (Fig. S3).

Squamous epithelial cells are frequently seen in women, either from vaginal contamination or from squamous metaplasia. Cylindrical epithelial cells from the trigonum commonly appear in catheterized urine samples, usually in clusters. Renal tubular cells are small and cuboidal in shape (Fig. S4). Seminal vesicle cells contain cytoplasmic pigment and represent male genital contamination. Urethral gland cells are columnar with vesicular nuclei and mucin-containing vacuoles; these may proliferate under estrogenic influence.

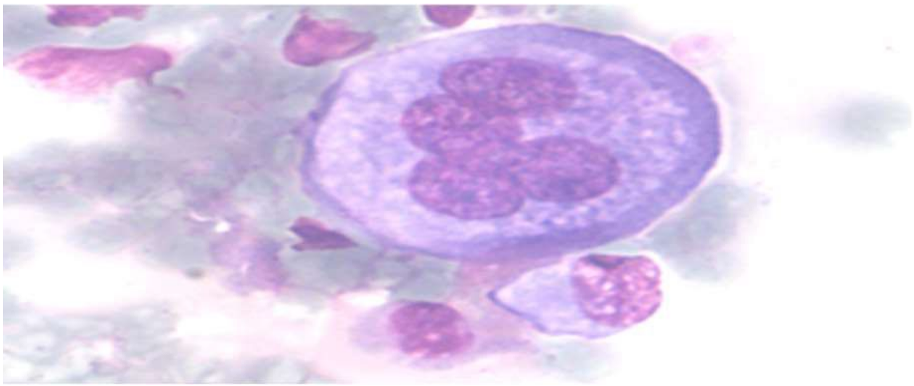

Figure S1. Superficial cells (umbrella cells) (MGG, obj., × 40).

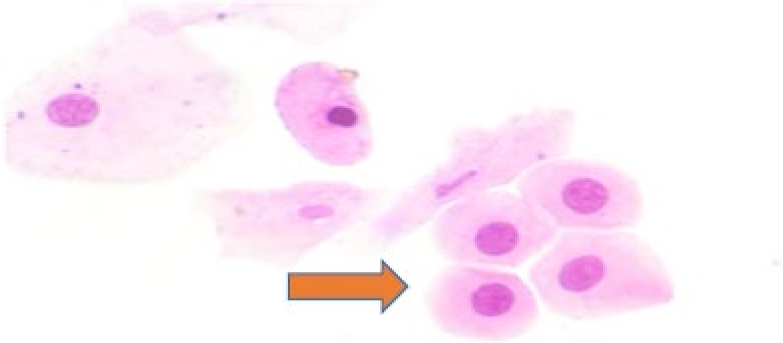

Figure S2. Intermediate urothelial cells (Papanicolaou, obj., × 20).

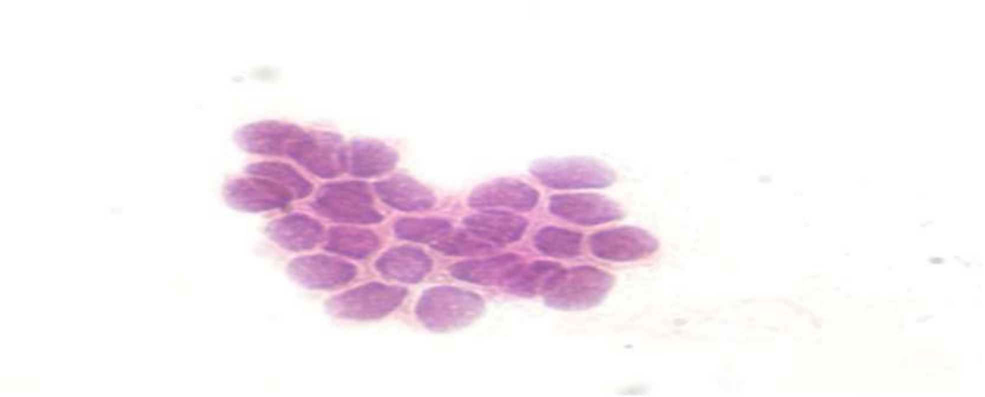

Figure S3. Basal urothelial cells (Papanicolaou, obj., × 40).

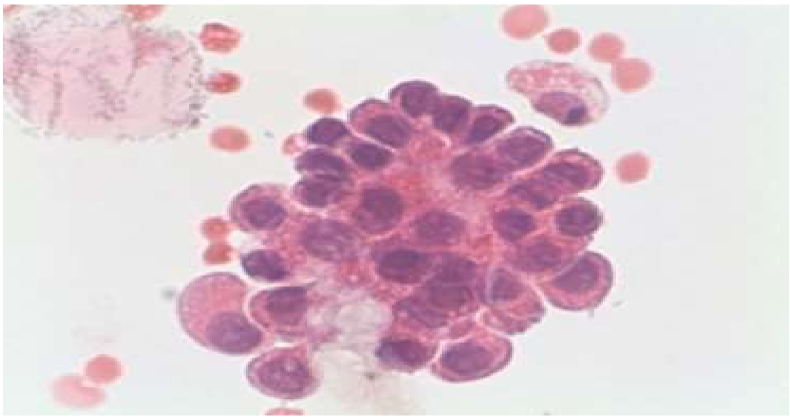

Figure S4. Renal tubular epithelial cells (Papanicolaou, obj., × 20).

Notably, nucleoli are generally absent in cells from spontaneous urine, and cellular aggregates are uncommon. Contaminants include vulvar squamous cells (women), rarely prepucial cells (men), spermatozoa, and seminal vesicle cells—often with pyknotic nuclei and granular cytoplasm—which may mimic neoplastic cells. Additionally, bacterial or fungal colonization can complicate cytological interpretation.

2. Cytomorphological Appearance of Reactive and Inflammatory Changes

Mechanical Irritation (Catheterization, Irrigation, Urolithiasis): Mild nuclear enlargement and cytoplasmic vacuolization.

Metaplasia:

Squamous metaplasia: Physiological in women, especially in the trigonum.

Glandular metaplasia: Secondary to chronic irritation or inflammation.

Post-Radiotherapy: Urothelial cells display nuclear swelling and cytoplasmic vacuolization (Figs. S5, S6).

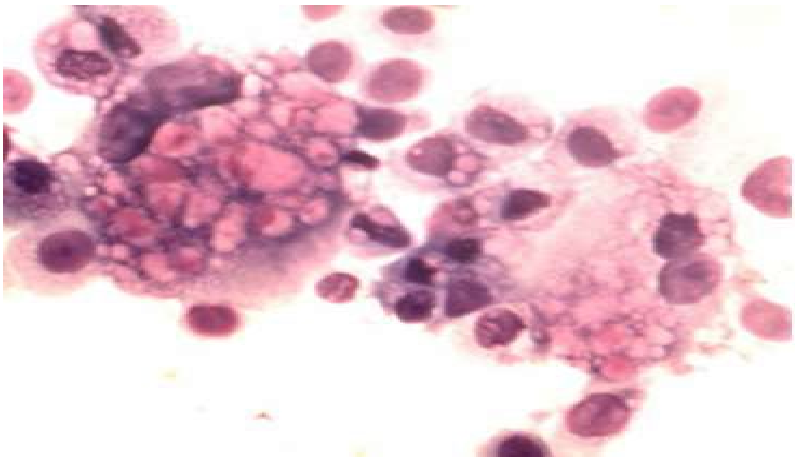

Figure S5. Macrophages and Urothelzellen with cytoplasmic vacuoles after administration of contrast medium (Papanicolaou, obj., × 20).

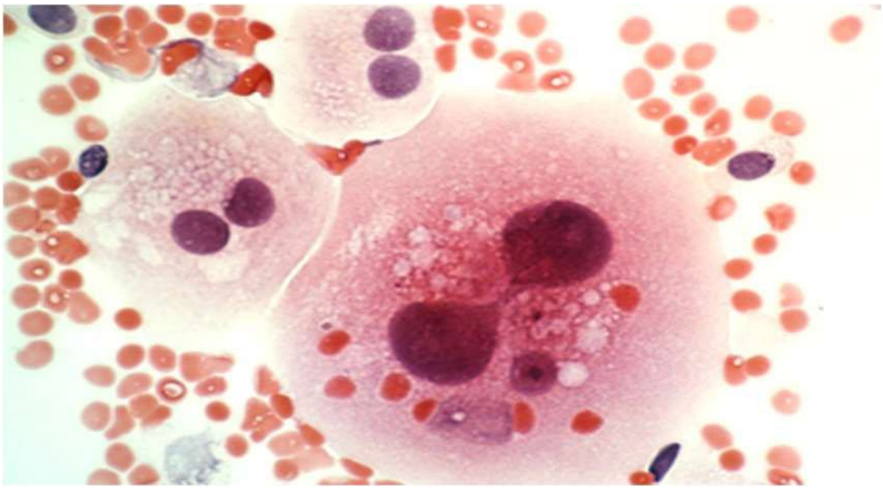

Figure S6. Urothelial changes after radiotherapy (Papanicolaou, obj., × 40.

Infections:

Bacterial: Presence of neutrophils and reactive urothelial cells.

Viral (e.g., SV40, CMV): Intranuclear inclusions (Fig. S7A, B).

Ileum Conduit: Abundant cylindrical epithelial cells, often with debris (Fig. S8A, B).

Cytostatic Agents:

Mitomycin, BCG: Atypical nuclei in urothelial cells with cytoplasmic maturation; may mimic malignancy (Fig. S9).

Azathioprine: Nuclear enlargement, hemorrhage, eosinophilic cytoplasm, minimal inflammation.

Urothelial Hyperplasia: Flat or papillary, defined as at least seven cell layers; suggestive in cytology, often linked to polypoid cystitis.

Uric Acid Crystals: Associated with bleeding and inflammation; clusters of reactive epithelial cells with atypia.

Non-Specific Cystitis: More common in women; presents with inflammatory urothelial changes.

Papillary, Polypoid, Bullous Cystitis: Variable epithelial and inflammatory changes.

Metanephric Metaplasia: Benign, small cuboidal cells without significant atypia.

Urocystitis Cystica et Glandularis: Glandular metaplasia with cystic changes (Fig. S10A, B).

Malakoplakia: Macrophages with Michaelis–Gutmann bodies (Fig. S11).

Inflammatory Pseudotumor: Spindle cell nodules post-surgery, mimicking neoplasia.

Cytology of Inflammation: Neutrophils, erythrocytes, and lymphocytes indicating active inflammation (Fig. S12).

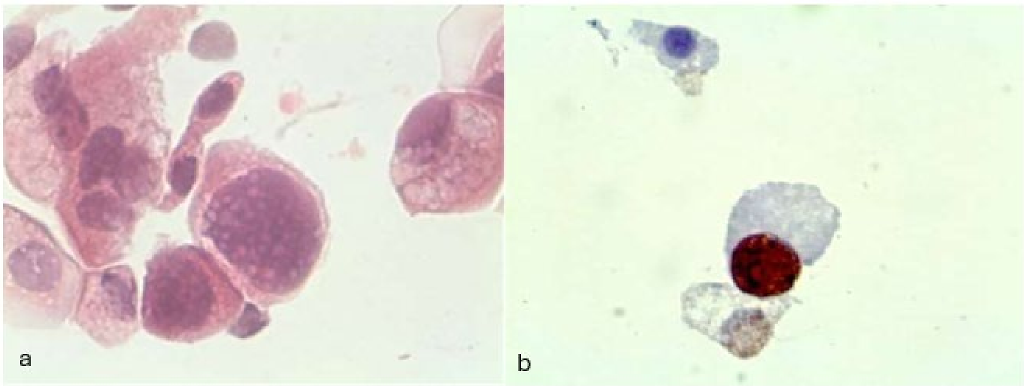

Figure S7. a: Decoy cells by Polyomavirus (Papanicolaou, obj., × 40). b: Immunohistochemistry: SV40-positive (obj., × 20).

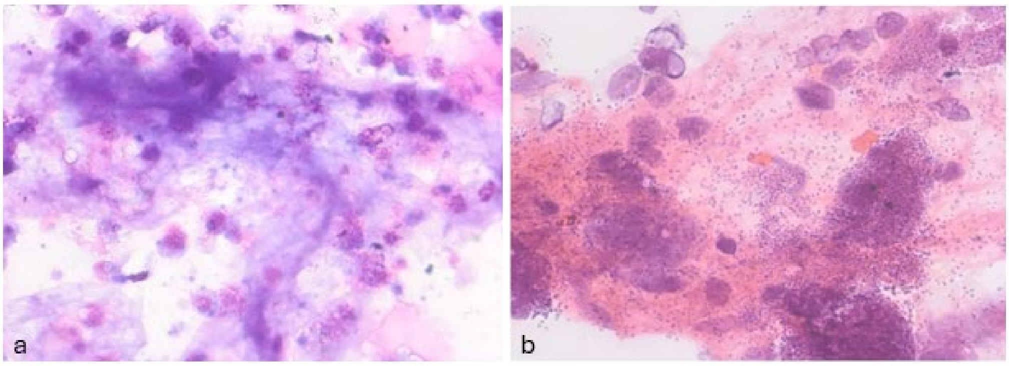

Figure S8. a, b: Conduit urine (Papanicolau, obj., × 10).

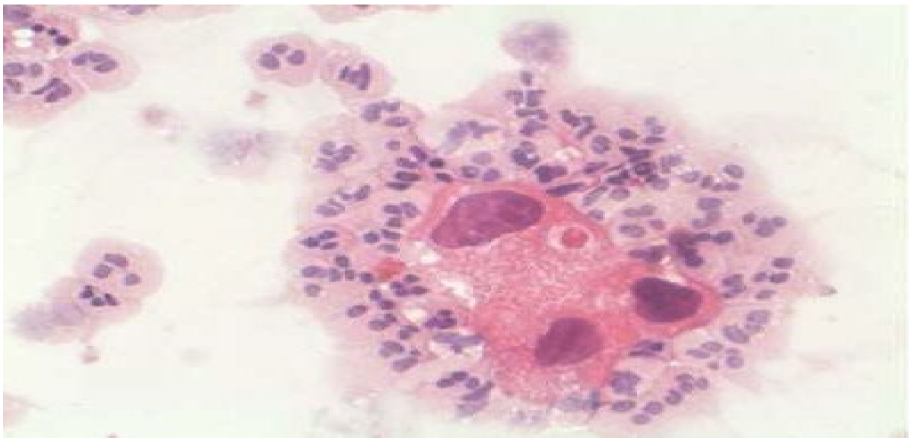

Figure S9. Urine changes after BCG therapy (Papanicolaou, obj., × 20).

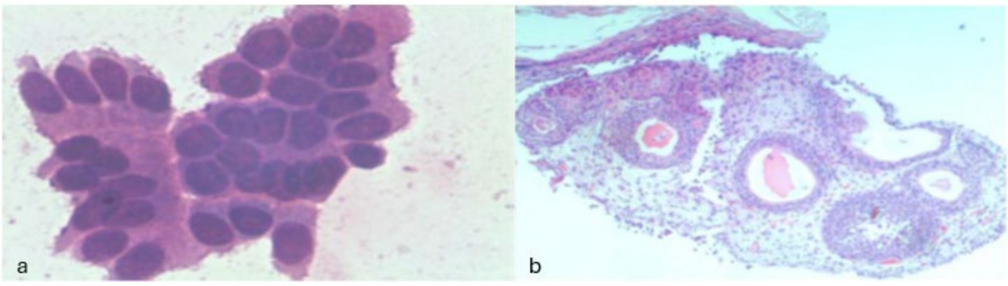

Figure S10. a, b: Urocystitis cystica et glandularis Brunn's cell nests (a: Papanicolau; b: HE obj., × 40/obj., × 10).

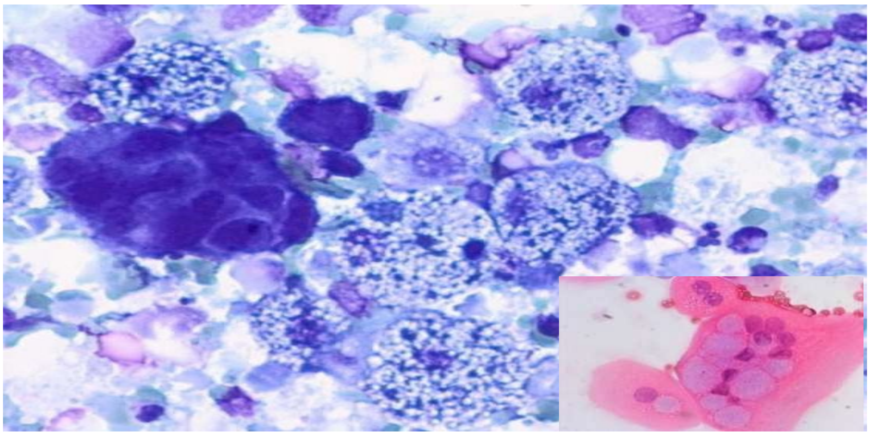

Figure S11. Malakoplakia (MGG obj., × 40).

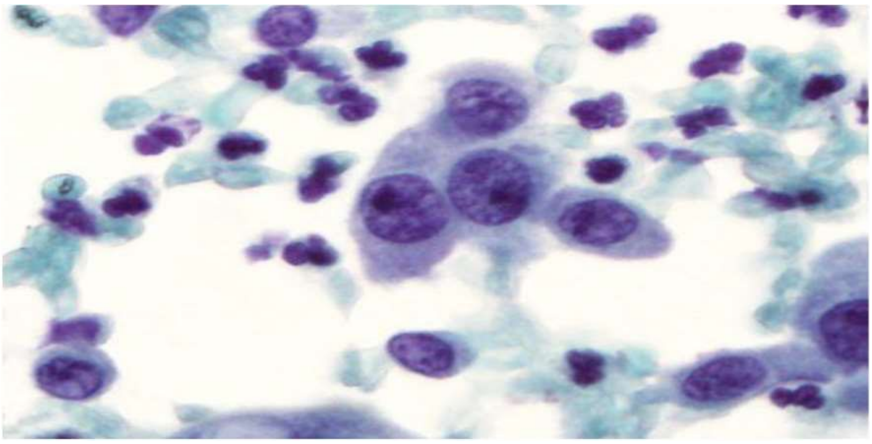

Figure S12. Inflammatory changes of urothelial cells (MGG, obj., × 40).

3. Cytomorphological Appearance of Atypical, Dysplastic, and Neoplastic Changes

Atypia of Unclear Significance: Hyperchromatic nuclei with eosinophilic nucleoli; increased N/C ratio; normal cytoplasmic maturation; low mitotic activity. Features may mimic reactive or low-grade neoplastic changes, requiring further clinical correlation.

Flat Neoplasms:

Urothelial Dysplasia: Isolated or clustered cells with hyperchromatic, polymorphic nuclei; coarse chromatin; mature, cyanophilic cytoplasm; elevated N/C ratio (Fig. S13A–C).

Carcinoma in situ: Highly pleomorphic cells with hyperchromatic nuclei, prominent nucleoli, sparse cytoplasm, and frequent mitoses (Fig. S14).

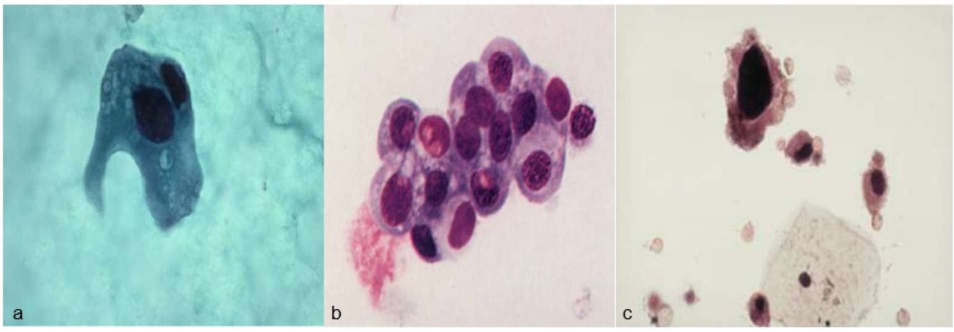

Figure S13. a, b, c: Urothelial dysplasia (Papanicolaou, obj., × 20).

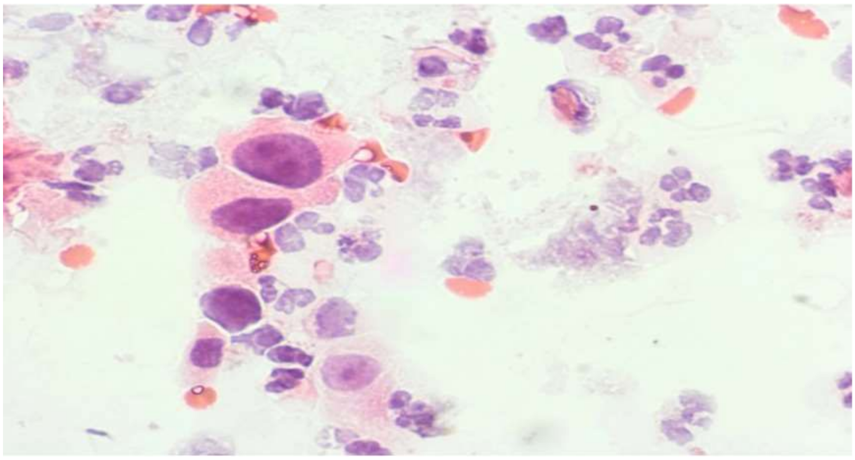

Figure S14. Urothelial carcinoma in situ (CIS) (Papanicolau, obj., × 20).

Benign Papillary Neoplasms:

Papilloma: Rare; small cohesive groups of cubic cells; mildly enlarged nuclei; no nucleoli or atypia (Fig. S15).

Inverted Papilloma: Cell clusters without significant atypia or nucleolar prominence; diagnosis often presumptive.

PUNLMP: Previously classified as G1 papillary carcinoma in the 1973 WHO classification [6]; mild atypia, low N/C ratio.

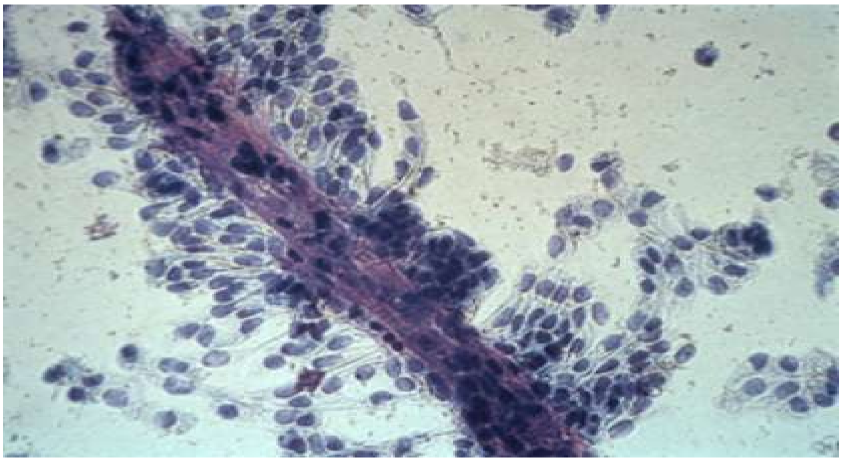

Figure S15. Papilloma (MGG, obj., × 20).

Malignant Papillary Neoplasms:

Low-Grade Non-Invasive Carcinoma: Cell-rich samples with hyperchromatic, oval or round nuclei; moderate nuclear enlargement; increased N/C ratio; absence of nucleoli; occasional mitoses (Figs. S16-S19).

High-Grade Non-Invasive Carcinoma: Highly cellular with grouped or isolated dysplastic cells; irregular, pyknotic nuclei; immature, vacuolated cytoplasm; N/C ratio >0.7; bloody background (Figs. S18, S21).

Invasive Carcinoma: Predominantly high-grade urothelial origin. Major risk factors include smoking, exposure to aromatic amines, and chronic infection (e.g., schistosomiasis). Recurrence is common; prognosis depends on tumor stage. Cytology cannot determine invasiveness (Figs. S22-S24).

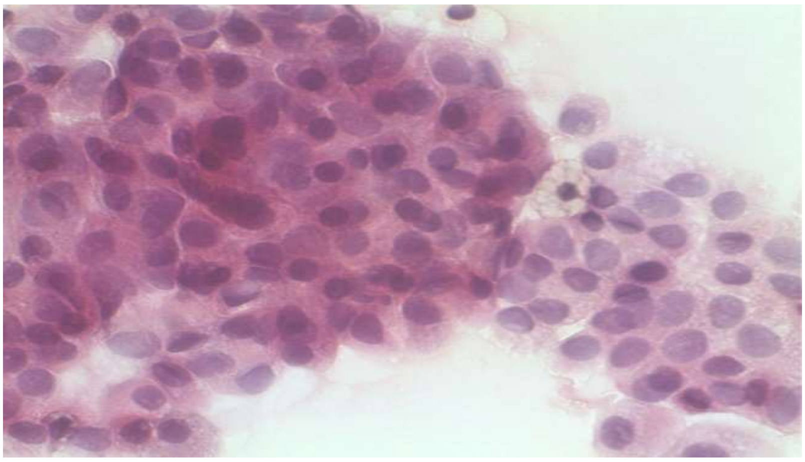

Figure S16. Urothelial carcinoma, G1 (Papanicolau, obj., × 20/× 40).

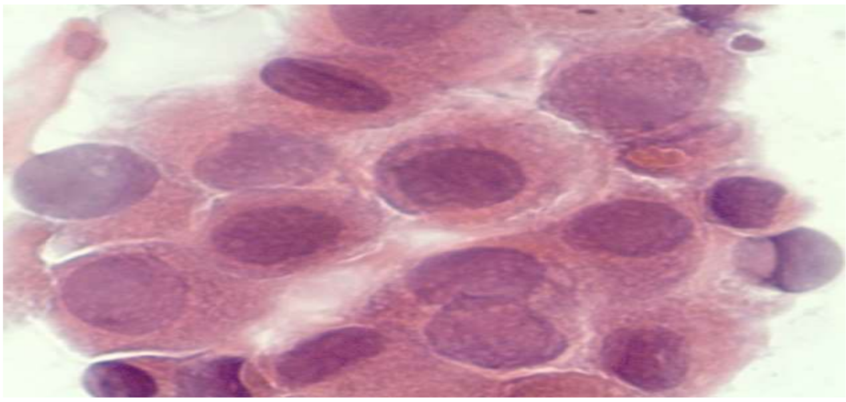

Figure S17. Urothelial carcinoma, G2 (Papanicolau, obj., × 40).

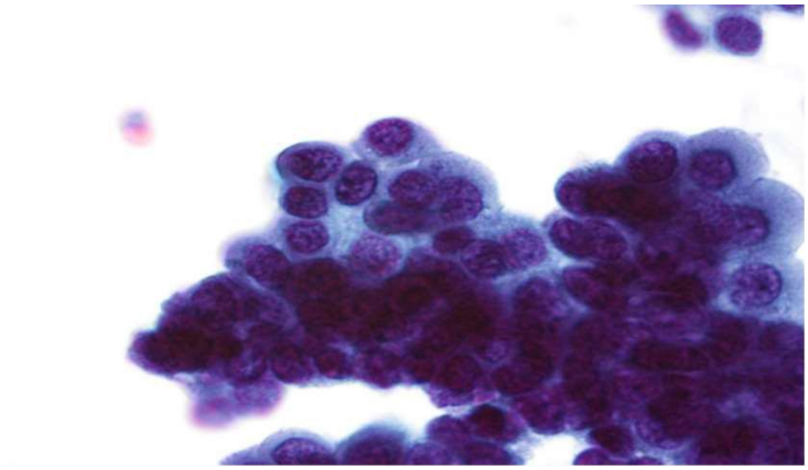

Figure S18. Low-grade urothelial neoplasma (MGG, obj., × 20).

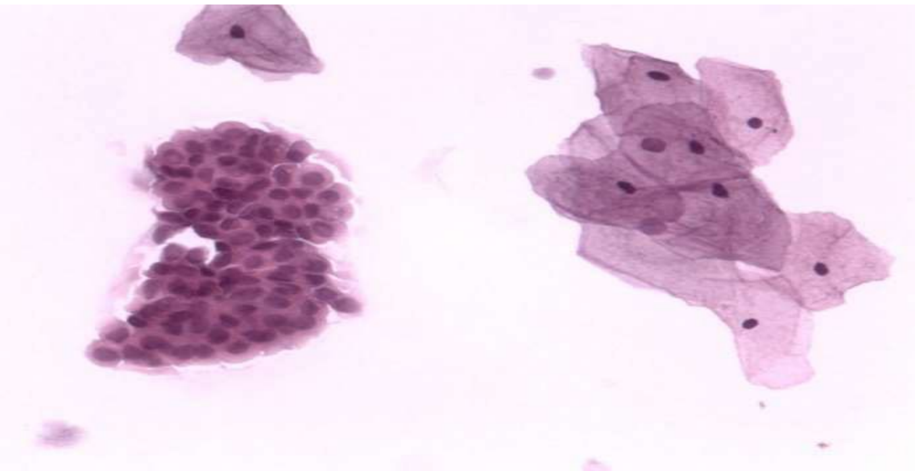

Figure S19. Spontaneous urine, papillary low-grade urothelial neoplasma, G1 (Papanicolau, obj., × 20).

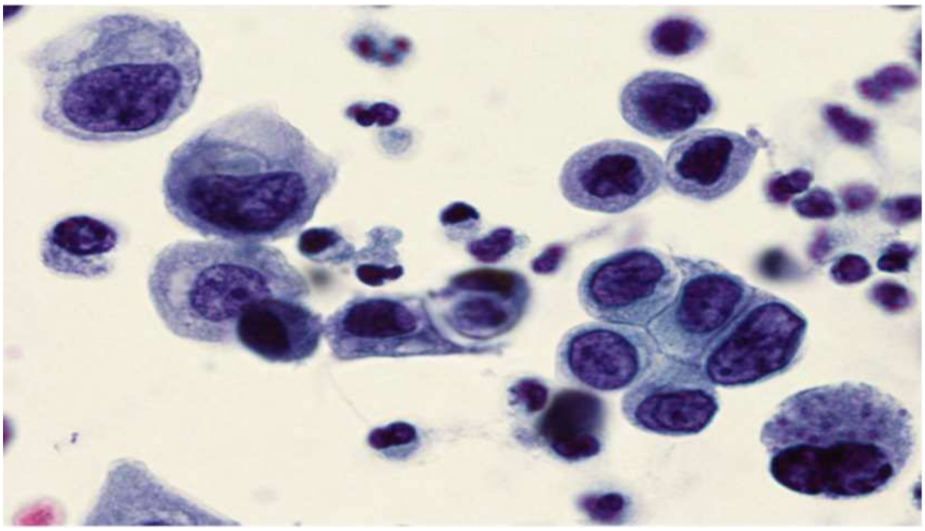

Figure S20. UB ca., G3 (MGG, obj., × 20).

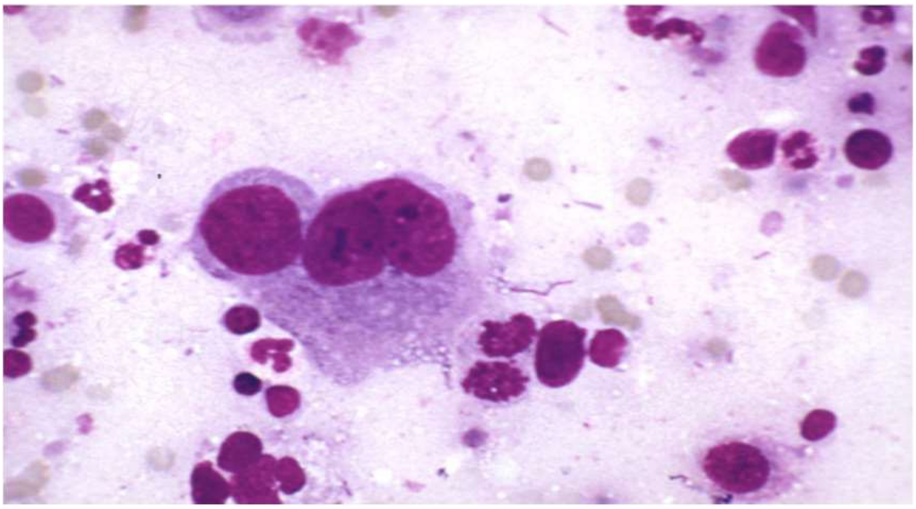

Figure S21. Urothelial carcinoma, G2 (Papanicolau, (obj., × 40).

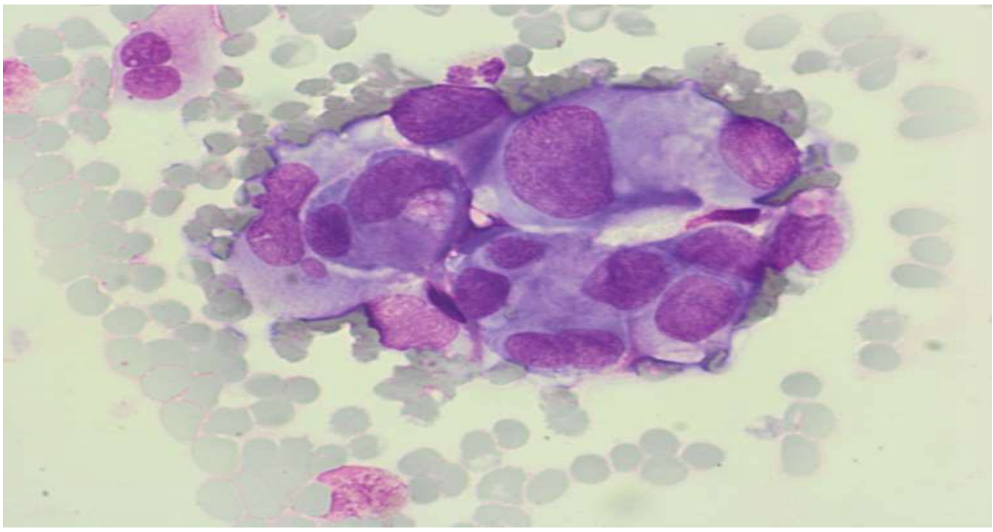

Figure S22. Urothelial carcinoma of the bladder (Papanicolaou, obj., × 40).

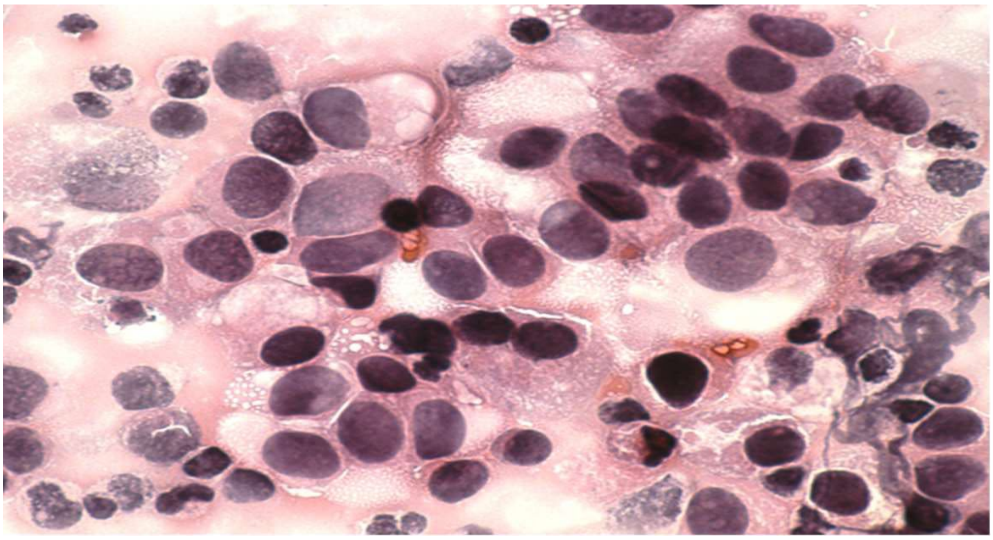

Figure S23. Urothelial carcinoma, G3 (Papanicolau, (obj., × 20).

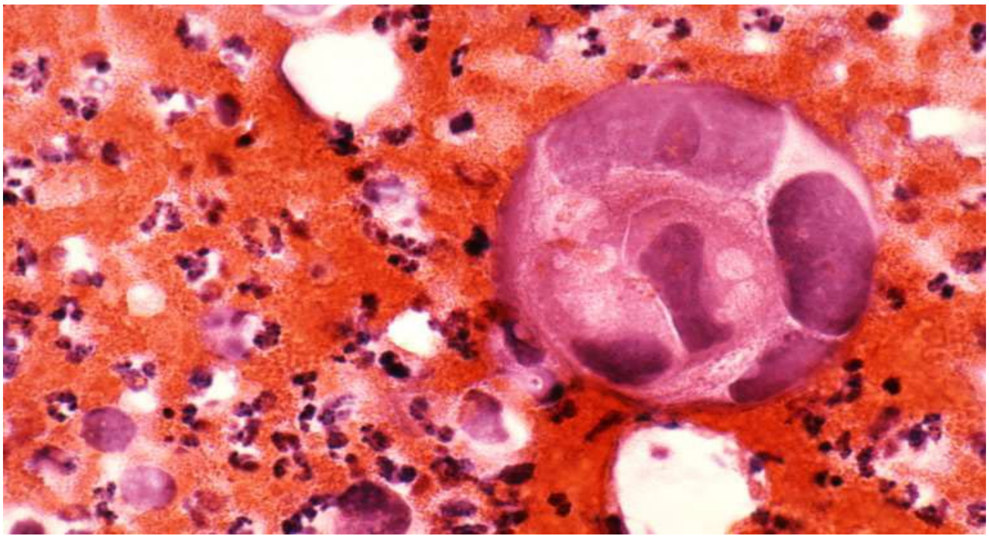

Figure S24. Urothelial carcinoma, G3 (Papanicolau, (obj., × 20).
